# Impact of a Gluten-Free Diet in Adults With Celiac Disease: Nutritional Deficiencies and Challenges

**DOI:** 10.7759/cureus.74983

**Published:** 2024-12-02

**Authors:** Monther Ghunaim, Alaa Seedi, Dalia Alnuman, Shouq Aljohani, Nihal Aljuhani, Mayar Almourai, Shahad Alsuhaymi

**Affiliations:** 1 Department of Internal Medicine, Taibah University, Al-Madinah, SAU

**Keywords:** celiac disease, challenges with gfd, gluten free diet, gluten free products, nutritional deficiencies, quality of life

## Abstract

Celiac disease (CD) is a long-term inflammatory condition affecting the small intestines, characterized by bowel villi atrophy and mucosal histological alterations that lead to impaired nutrient absorption and metabolic changes. While a gluten-free diet (GFD) is recognized as one of the most effective treatments, it presents significant challenges including increased expenses, potential nutritional deficiencies, and various social and psychological implications. This review evaluates the comprehensive impact of GFD on CD patients, examining its efficacy in preventing complications like osteoporosis and alleviating symptoms, while also addressing the difficulties in maintaining complete gluten elimination. Although many individuals experience symptom resolution and recovery through dietary adherence, the restrictive nature of GFD can lead to nutritional deficiencies. Furthermore, patients often face psychological challenges including anxiety, fear, sleep disturbances, and other mental health concerns while maintaining the diet. This review synthesizes the current understanding of both the therapeutic benefits and multifaceted challenges associated with GFD adherence in CD management, providing insights into its effects on patients' physical health and quality of life.

## Introduction and background

Celiac disease (CD) is an autoimmune condition triggered by dietary gluten in individuals with a genetic predisposition, resulting in histological alterations in the small intestine, and over the last 20 years, CD has become a significant global public health issue [1]. A comprehensive study involving 275,818 participants estimated the global prevalence of CD to be 1.4% [2]. Patients with CD are sensitive to gluten, a specific protein composite found in wheat, and gluten consists of water-insoluble prolamins, which are a group of alcohol-soluble lectins that are key seed proteins in cereal grains. Glutamine and glutenin, the most abundant prolamins in wheat, are primarily responsible for triggering symptoms in CD [3]. Ingestion of gluten initiates a reversible inflammatory reaction in the small intestinal mucosa, presenting with acute symptoms like diarrhea, constipation, nausea, and vomiting, and prolonged exposure to gluten can lead to chronic mucosal damage and inflammation, resulting in nutrient malabsorption, including deficiencies in calcium, vitamin D, iron, vitamin B12, folic acid, and zinc [4-6]. Such deficiencies can cause severe outcomes like anemia, osteoporosis, and impaired growth [7]. While adults may experience milder gastrointestinal symptoms, severe diarrhea is a predominant complaint in many cases [8]. Currently, the primary treatment for CD is medical nutrition therapy through a strict gluten-free diet (GFD) [9]. Effective management also involves monitoring nutritional status via blood tests and supplementing gluten-free nutrients as necessary, and optimal GFD should consist of naturally gluten-free, nutrient-rich foods that are affordable, accessible, and balanced in macro- and micronutrients. Incorporating pseudo-cereals into the diet provides an excellent source of complex carbohydrates, proteins, fiber, essential fatty acids, vitamins, and minerals [10].

## Review

The study aims to determine the efficacy of a GFD among CD patients, evaluate the impacts of nutritional deficiencies arising from a GFD, and assess the psychological impacts and challenges that may interfere with CD.

Efficacy of GFD and cross-contamination

CD is a long-term autoimmune condition affecting the small intestine, and it is characterized by a gradual deterioration of the intestinal villi following the ingestion of gluten [11]. The significance of enhancing and expanding CD diagnosis has been established for a long time, and currently, there is a growing population of individuals diagnosed with CD who are adhering to a GFD [12]. The GFD is a useful diet for preventing problems including osteoporosis and malabsorption, as well as gastrointestinal and extra-intestinal symptoms [13,14]. Current guidelines recommend that the GFD should be strict, requiring complete avoidance of gluten-containing products and careful attention to cross-contaminations [15]. Even when strictly following a GFD, it is impossible to completely eliminate gluten, as many food items, including sausages, soups, soy sauce, and ice cream, may contain hidden traces of gluten; furthermore, products labeled as "gluten-free" can still have small amounts of gluten due to cross-contamination during processing or storage with gluten-containing items, meaning that the term "gluten-free" generally refers to an amount of gluten that is considered safe and does not mean the total absence of gluten. In fact, the gluten content resulting solely from cross-contamination can potentially vary from 5 to 50 mg per day [16,17]. Kurppa et al. conducted a study comparing the effectiveness of a GFD in patients with mild enteropathy and those with villous atrophy, finding that the GFD yields comparable results in terms of mucosal architecture recovery, reduction of intestinal mucosal inflammation, antibody concentrations, and improvement of symptom [18]. Approximately 34% of adult patients achieve mucosal recovery within two years, whereas within five years, around 66% experience this recovery. Nonetheless, research indicates that older patients, specifically those between 30 and 60 years old, may have incomplete recovery, and individuals above 60 years old show no statistically significant signs of recovery [19]. A small proportion of individuals (approximately 0.04-1.5%) suffer from refractory CD, which is characterized by ongoing symptoms and villous atrophy despite strict adherence to a GFD [20,21]. Refilling nutrient levels and ensuring sufficient nutrient intake in individuals with CD continues to be a difficult task; however, among CD patients following a GFD, certain nutrients such as vitamin B12, folic acid, calcium, and magnesium seem to be generally restored. It has been shown that vitamin B12 deficiency is not observed in treated adult CD patients [22]. Approximately 14% to 41% of adult celiac patients following a GFD have been reported to experience iron deficiency [23]. Due to the intestinal mucosal repair during the first year of GFD, anemia was significantly improved in the majority of individuals (but not all). Additionally, women's recovery seemed to be slower than men's based on menstrual blood loss, which was looked to be the reason. Therefore, for patients who are strict with GFD, foods high in iron might be advised, and as part of CD care in clinical settings, supplements should be provided [24]. Inadequate bone health can have long-lasting effects on an individual’s well-being, and therefore implementing additional measures to enhance bone health and prevent further bone loss in CD patients following a GFD could be beneficial. One potential treatment option that is being considered is the use of calcium and vitamin D supplements, because these supplements have the potential to improve bone health in CD patients who are on a GFD [25]. In addition, vitamin B12 supplements may help prevent neurological complications, while folic acid supplementation has been shown to reduce anxiety and depressive symptoms in long-standing CD cases. A six-month regimen of folic acid supplementation can significantly improve mood and overall well-being [26,27].

Nutritional Deficiencies Among Celiac Patients Following a GFD

Lifelong adherence to a GFD is the cornerstone of treatment for individuals diagnosed with CD. Non-compliance with the diet can exacerbate symptoms, lead to further intestinal damage, and significantly increase the risk of certain cancers. For instance, the risk of small intestinal adenocarcinoma is 4-10 times higher in individuals with CD compared to healthy individuals, while esophageal cancer, melanoma, and non-Hodgkin’s lymphoma also show elevated risks, with approximately 2-3% of CD patients being affected [28]. Because gluten is found in common grains like wheat, barley, rye, and oats, adhering to a GFD means completely avoiding these foods. To maintain a balanced diet, patients are encouraged to incorporate alternatives like fruits, vegetables, fish, meat, and gluten-free products (GFPs) [28]. Studies indicate potential nutritional deficiencies in the first year of a GFD due to the strict diet until the mucosal recovery occurs. Once this happens, diet can be modified, and supplements may be prescribed [27]. However, both macronutrient and micronutrient imbalances are still common among CD patients. Deficiencies and distribution can be seen in both macronutrients and micronutrients.

Macronutrient intake

Fats

The unbalanced consumption of fats among individuals with CD has been consistently confirmed across various studies [25]. While some research focuses on highlighting high fat intake, others also emphasize notable consumption of cholesterol or saturated fatty acids (SFA) [26,27]. This imbalance may be attributed to the frequent consumption of processed GFPs combined with a limited intake of plant-based foods. It is common for individuals with CD adhering to a GFD to consume GFPs, which often have a higher content of total fats and SFAs compared to their gluten-containing equivalents. For example, gluten-free bread may contain up to 20% more fat than regular bread [26]. Diets that are disproportionately high in SFAs are associated with health issues such as insulin resistance (IR) and an increased risk of cardiovascular diseases (CVD) in both the general population and the celiac population. Notably, individuals with CD have been observed to face a higher risk of mortality due to CVD. Additionally, the consumption of processed foods has been strongly linked to increased mortality rates [28-30]. Chronic low-grade inflammation, which is frequently associated with unbalanced diets characterized by these features, is another significant concern.

Carbohydrate

The most popular cereals are gluten-containing cereals, which must be avoided when starting a GFD for individuals with CD [29,31]. Numerous studies have indicated that individuals following a GFD tend to consume fewer carbohydrates, potentially due to their avoidance of cereal products out of fear of inadvertently consuming gluten. While overall carbohydrate intake among GFD patients may appear sufficient, much of it comes from simple sugars and processed foods with a high glycemic index. For example, gluten-free cookies and pastries often have a glycemic index 15-25% higher than their traditional counterparts. This reliance on high-GI foods can increase the risk of IR and metabolic disorders over time [32,33]. This condition is associated with a greater prevalence of hyperinsulinemia, reduced glucose tolerance, and an increased risk of diabetes.

Fibers

Most studies show that fiber intake is low in CD patients on a GFD, reflecting their lower consumption of carbohydrates and high-fat consumption [34]. This lack of fiber is linked to gastrointestinal issues like constipation, diverticulitis, and persistent abdominal discomfort. Additionally, it has been connected to a higher risk of gastrointestinal symptoms that are frequently seen in CD and even in people with treated CD [34,35]. It is possible that people with increased fiber intake may experience fewer symptoms such as abdominal pain and an improvement in the disease's visible inflammation.

Proteins

Research indicates that individuals on a GFD tend to consume more protein than needed, primarily from meat. While this may not pose an immediate problem, a balanced intake is still advised [27,35].

Micronutrient intake

Regarding micronutrients, the research indicates that the consumption of vitamins and minerals is also impaired.

Vitamins

Vitamin deficiencies are quite common among CD patients, especially for vitamins D and E, as well as B-group vitamins like folate (B9), thiamine (B1), riboflavin (B2), and pyridoxine (B6) [23,27,36,37]. Vitamin D deficiency is particularly concerning because of its role in bone health, with low levels contributing to the higher rates of osteoporosis seen in CD patients. As a result, supplementation with 1,000-2,000 IU of vitamin D daily is often recommended during the first year of treatment [36,38]. B-group vitamin deficiencies are also significant, with studies reporting low levels of B12 and folate in biochemical assessments. The study noted elevated total homocysteine (tHcy) levels in these patients, despite long-term adherence to a GFD. Elevated tHcy levels, associated with an increased risk of CVD, underscore the importance of proper dietary treatment and regular monitoring [23,28,39,40].

To address this issue, it's crucial to emphasize that the mentioned deficiencies are linked to inadequate intake rather than intestinal malabsorption. Additionally, supplementing B-group vitamins has been observed to normalize elevated tHcy levels. Therefore, proper dietary treatment and regular monitoring are crucial. Di Nardo et al.'s study highlights the significance of folate intake, suggesting the consumption of pseudo-cereals like quinoa and amaranth, in addition to the typical sources found in vegetables and pulses. Moreover, low B-group vitamin levels are linked to a lower quality of life, and supplementation is associated with improved overall well-being [39].

Minerals

Common mineral deficiencies in CD patients include iron, calcium, magnesium, iodine, potassium, and zinc, though deficiencies in selenium, sodium, and manganese have also been reported [26,31,37,41]. While a suitable GFD can normalize deficiencies like zinc, it may not be sufficient for others, such as magnesium, as GFPs have lower mineral content than their gluten-containing counterparts. Iron deficiency is particularly critical in untreated CD and in patients with incompletely regenerated mucosa, with difficulties in achieving normal iron values [42]. Hallert et al. observed no serum iron deficiencies after 10 years. Iron deficiency is a common complication of CD, especially in women, and can worsen due to low consumption of legumes and cereals. Special attention is needed for these deficiencies, considering them alarming, particularly in women [39,42]. As mentioned, while a GFD is essential for managing CD, addressing nutritional deficiencies requires ongoing monitoring, dietary adjustments, and sometimes supplements to ensure long-term health and well-being [43].

Challenges in adhering to a GFD

CD is a condition that has long been associated with various neurological and mental health disorders, including epilepsy, dementia, and depression [44]. The impact of dietary restrictions associated with CD can lead to significant feelings of isolation and dissatisfaction within social contexts, as food often plays a central role in cultural traditions and gatherings [1]. To better understand the implications of CD, a qualitative cross-sectional study was conducted. This study aimed to gather information on the symptoms associated with CD and explore the effects of these symptoms, as well as adherence to a GFD on the daily lives and functioning of patients [45]. The themes explored encompassed fears and anxiety, the daily management of CD, physical functioning, sleep patterns, daily activities, engagement in social activities, emotional well-being, and the dynamics of relationships [45]. A study conducted by Scarff [46] highlighted the potential development of orthorexia nervosa as a consequence of strict adherence to a GFD. Each of these psychological impacts will be discussed separately.

Orthorexia Nervosa and Strict Adherence

Strict adherence to dietary restrictions has been found to have a negative impact on the quality of life among adolescents. Although not officially classified in the Diagnostic and Statistical Manual of Mental Disorders, there is a concerning phenomenon known as orthorexia nervosa, which involves an obsessive preoccupation with restrictive eating. This phenomenon is particularly concerning among individuals who follow a GFD [1]. Orthorexia nervosa refers to the behavior of individuals who, despite lacking a medical necessity (as they have a healthy weight and no diagnosed medical condition), engage in increasingly restrictive diets. This behavior can adversely affect both health and quality of life [44]. In a study conducted by Wolf et al. [47], it was observed that individuals exhibiting a high level of vigilance in complying with a GFD encountered a diminished quality of life due to increased anxiety levels. This elevated anxiety poses a potential susceptibility to the development of orthorexia nervosa, as these individuals remain highly focused on their gluten-free dietary practices [1].

Anxiety and Fears

Participants commonly conveyed feelings of apprehension and anxiety following their diagnosis of CD. Their concerns largely centered on the possibility of inadvertently consuming gluten, which could trigger symptoms. Participants also expressed fears about the prospect of passing the disease on to future generations and the long-term consequences of the CD. Specifically, there were reports of unease when consuming meals outside the home due to the fear of unintentionally consuming gluten and encountering symptoms [44]. Female participants, particularly those with children, shared concerns about the hereditary transmission of CD to their offspring. One participant mentioned her child's already-diagnosed condition, prompting ongoing worries about the long-term implications for her child. Furthermore, participants expressed anxieties regarding potential complications and the heightened risk of cancer associated with CD [45].

Health-related quality-of-life (HRQOL) impacts

The study conducted by Leffler et al. [45] also focused on the impact of CD on HRQOL, delving into various aspects of this impact discussed separately.

Sleep

Symptoms have an impact on sleep patterns, with patients reporting disruptions in their sleep patterns, often waking at night due to symptoms such as abdominal pain. Conversely, some individuals experienced increased fatigue, which led them to require additional sleep [45].

Physical Activities

Study participants conveyed those symptoms of CD had adverse effects on their engagement in physical activities. More specifically, symptoms such as fatigue, headaches, and joint pain imposed limitations on participants' involvement in sports or exercise. A notable instance was shared by one participant who mentioned that the occurrence of flatulence prevented her from attending the gym [45].

Daily Activities

Numerous symptoms reported by participants adversely influenced their day-to-day activities. For instance, the occurrence of diarrhea compelled participants to remain close to a bathroom, restricting their involvement in specific daily activities. Symptoms such as abdominal pain, nausea, headaches, or muscle and joint pain impeded participants' ability to perform routine tasks such as grocery shopping or household chores. Moreover, various symptoms disrupted participants' professional or academic pursuits. Cognitive symptoms led to missed appointments and hindered effective work, while bloating affected clothing fit and dictated the attire participants could comfortably wear [45].

Social Activities

Various symptoms of CD exert an influence on social activities. Gastrointestinal symptoms, such as constipation, abdominal pain, nausea, and gas, frequently disrupted the enjoyment of social activities or led participants to abstain from social events altogether. Additionally, fatigue reduced participants' inclination to engage in evening outings or make social plans. Conforming to a GFD also negatively affected social activities. Participants expressed challenges in attending food-related social events, particularly dining out at restaurants. These difficulties arose from concerns about the availability of gluten-free food and uncertainties about whether meals were prepared in a gluten-free kitchen. Consequently, participants occasionally refrained from social outings for these reasons [45]. Silvester et al. [48] conducted a study that provided additional evidence of the social isolation linked to the GFD. The research revealed that CD patients who were non-strict in following a GFD occasionally consumed gluten intentionally. Interestingly, this group reported experiencing greater enjoyment and lower levels of anger and depression compared to CD patients who adhered more strictly to the diet. The latter group experienced a higher degree of social isolation, often choosing to eat at home rather than in public spaces. These findings underscore the psychological and sociological challenges associated with maintaining a GFD [1].

Strict adherence to dietary restrictions has been found to negatively impact the quality of life among adolescents. Participants conveyed a range of emotional effects associated with experiencing symptoms of CD. Depression and anxiety were frequently linked to symptoms such as diarrhea, abdominal pain, fatigue, and cognitive issues. The emotional impact extended to other negative feelings, including anger, annoyance, distress, and irritability arising from the symptoms. Adhering to a GFD also contributed to negative emotions. Participants often expressed feelings burdensome or bothersome to others who had to accommodate their dietary requirements, diminishing the enjoyment of social activities. Additionally, the necessity to disclose their disease to others was described as awkward and uncomfortable at times [45]. For certain participants, the inability to engage in or derive enjoyment from activities due to symptoms had an adverse impact on their relationships with family and friends. While some patients noted understanding and support from friends and family, others experienced annoyance, which negatively affected their relationships. Participants expressed a sense that some in their social circle lacked comprehension of their disease. The planning required for GFD adherence and ensuring a constant supply of gluten-free food also posed challenges for participating in spontaneous activities with friends or family [45]. Adhering to a stringent GFD presents challenges, especially for specific demographic groups such as the elderly, individuals with limited literacy, those with mental or psychological impairments, and those facing financial restrictions. Hence, it is advisable to recommend a GFD only after confirming the diagnosis of CD through serological tests and examination of duodenal histology [49]. The results of the study by Leffler et al. indicate that both experiencing symptoms and the necessity of following a GFD have adverse effects on HRQOL [45]. Recent research has indicated that adults diagnosed with CD continue to face persistent symptoms and burdens associated with treatment, posing significant challenges. It is critical to closely monitor the quality of life and adherence to a GFD, and provide patient education on potential risks for individuals with CD who are following the diet [1]. As a result, there exists an evident gap in meeting the needs of the celiac population, and the objective of enhancing the quality of life for individuals with CD remains unfulfilled [45].

## Conclusions

GFD is considered the most well-known treatment for CD, and it is required for the patient to follow it for the rest of their lives. Adhering to a strict GFD presents several difficulties, such as low nutritional intake, increased expenses associated with strict adherence, and psychological obstacles. Consequently, there is a clear deficiency in addressing the requirements of those with CD, and the goal of improving their quality of life remains unachieved.
